# Notes and News

**Published:** 1903-02-14

**Authors:** 


### NOTES AND NEWS.

A Chinese hospital has been established in New York.

The study of tropical diseases has been stimulated in America by the creation of two professorships at the Michigan College of Medicine and Surgery.

Dr. Grünbaum has been appointed the first Director of the Cancer Research Institution, recently endowed by Mr. Sutton Timmis, of Liverpool, with £10,000.

There are a great many deaths from plague in the Bombay Presidency at the present time. The disease is largely prevalent elsewhere in India, but no district is so severely affected as Bombay.

Recent reports on the sanitation of Italy show a very deplorable array of statistics. In 600 communes the law providing a poor law doctor is disregarded. In 4,877 communities there are no drains and all refuse is thrown into the streets, and 336 communes have no burying place except the churches. The extreme poverty of the lower classes causes an immense amount of disease.

A new out-patients' department has been opened at the Liverpool Infirmary for Children. The whole of the present buildings are to be ultimately superseded. The new department consists of a large waiting-hall round which the various offices in connection with it are grouped. An ophthalmic room is provided, and an infectious room where patients can be housed without entering the main building.

In aid of the London Hospital Quinquennial Appeal, the students, past and present, of the London Hospital are organising a concert and dance at the Holborn Town Hall on February 17th. As the use of the Hall and the band have been granted free, the whole of the proceeds will go to the funds of the hospital. Tickets—double 10s. 6d., single 7s. 6d.—may be obtained of the Hon. Sec., Mr. W. H. Forshaw, London Hospital, E.

We learn that Mr. G. Q. Roberts, secretary and house governor of the London Hospital, has tendered his resignation.

The Lord Mayor will give a dinner at the Mansion House in aid of the Quinquennial Appeal of the London Hospital on May 4th.

Dr. J. W. Watkins, whose death has been announced, is understood to have been the first person to be anæsthetised by means of chloroform when the late Sir J. Simpson was experimenting in its use.

It appears probable that Bolingbroke House Pay Hospital, Wandsworth Common, will develop into a general hospital for South London. Bolingbroke House is well situated to serve a large locality, and the local appeal which will be made should meet with a good result.

New cures for cancer are constantly being announced. The latest specific advocated is that of molasses! Two cases are quoted from Queensland. The bad part of all such "remedies" is the waste of valuable time and the loss of the best chance of relief by surgical means which their use involves.

The Royal Naval Hospital at Stonehouse now shares with Haslar the distinction of training the naval sick-berth staff. Within a short time the numbers training at Stonehouse will be brought up to twenty-five. These probationers receive instruction in gymnastics, the general discipline of the Naval Service, and in sick cookery. The latter item of the curriculum will be superintended by the lady cook from Haslar.

The Assistance Publique of Paris has pasted up posters throughout the city against the use of alcohol in any form, whilst a counterblast is being circulated by the vendors of wine and other oponents, quoting the opinions of M. Duclaux, director of the Pasteur Institute, who declares that alcohol is a useful aliment. Public opinion is running very high on the subject, and the liquor trade is preparing to protect itself with vigour and money.

The report received by the Royal Society from their special commissioners who were despatched to Uganda to study the sleeping sickness so prevalent amongst the natives, is regarded so serious that they have obtained permission of the Secretary of State for War for Colonel Bruce, R.A.M.C., to proceed at once to superintend further investigations. Dr. Navarro, of the Pathological Laboratory of University College, will accompany him.

The late Mr. J. R. Wedgewood, of Barnes, has bequeathed £500 to the West London Hospital, and the late Hon. Mrs. G. Denman has bequeathed her house at Chislehurst to the London Hospital and the Poplar Hospital for the use of convalescent patients. She also provided in her will £500 for necessary alterations and repairs to the building. If the hospital authorities decide not to continue using the Home after three years or longer, it is to be sold for the benefit of both institutions.

